# Dual-Modal Chicken Mortality Detection Using Dynamic Hybrid Convolution-Based Feature Fusion

**DOI:** 10.3390/ani16071057

**Published:** 2026-03-31

**Authors:** Tian Hua, Qian Fan, Runhao Chen, Yulin Bi, Hao Bai, Zhixiu Wang, Guobin Chang, Wenming Zhao

**Affiliations:** 1 College of Animal Science and Technology, Yangzhou University, Yangzhou 225009, China; huatian0202@163.com (T.H.); ylbi@yzu.edu.cn (Y.B.); wangzx@yzu.edu.cn (Z.W.);; 2 College of Information Engineering, Yangzhou University, Yangzhou 225009, China; fanqian@yzu.edu.cn (Q.F.); crunhao@163.com (R.C.); 3 Joint International Research Laboratory of Agriculture and Agri-Product Safety, The Ministry of Education of China, Yangzhou University, Yangzhou 225009, China; bhowen1027@yzu.edu.cn

**Keywords:** dead chicken detection, dual-modal fusion, dynamic convolution, hybrid feature fusion, deep learning

## Abstract

In large-scale caged broiler farms, daily inspection of dead broilers is essential for flock health management and disease prevention. To address the significant performance degradation of existing methods under challenging conditions such as poor lighting, severe occlusion, and complex backgrounds, this paper proposes a dual-modal dynamic hybrid convolutional feature fusion method for dead bird detection based on an improved YOLO11 framework, termed YOLO11-DualDynConv-FF. First, a dual-modal fusion network architecture was developed to combine RGB and infrared (IR) images, enabling the model to simultaneously process both modalities. By integrating complementary information from RGB and IR data, the proposed method significantly improved detection accuracy and efficiency under low-light conditions. Second, a dynamic hybrid convolution feature fusion module was designed to merge multi-scale feature maps with contextual information, allowing the network to capture fine-grained details and adapt better to complex farming environments. In addition, an occlusion-aware module was introduced to specifically address the physical occlusion challenges prevalent in crowded cage settings. Comparative experiments and ablation studies involving multiple object detection networks were conducted to evaluate the proposed method. The results show that the improved YOLO11 model achieves superior performance, with precision, recall, F1-score, and mAP@0.5 reaching 92.6%, 79.0%, 0.85, and 80.1%, respectively. These results represent improvements of 2.0%, 5.0%, 0.17, and 12.1%, respectively, over the original YOLO11 model. Compared with existing approaches, the proposed model is better suited to complex real-world poultry farming environments and achieves higher detection accuracy, providing a valuable reference for intelligent monitoring in caged poultry farming.

## Simple Summary

Developing non-contact methods for dead broiler detection using computer vision technology can help improve the efficiency and accuracy of daily inspection in large-scale broiler farming. At present, dead broiler detection in complex farming environments still faces challenges such as low light, occlusion, and background interference. In this study, a deep-learning-based dual-modal detection method was proposed to improve the stability and reliability of dead broiler recognition by integrating image information from different sources. This method can provide technical support for intelligent monitoring and automated management in poultry farming and shows good application prospects.

## 1. Introduction

### 1.1. Research Background and Challenges in Poultry Farming

Chicken is the second largest source of meat in China after pork. In 2024, chicken consumption in China reached approximately 14.83 million tons, with a per capita consumption of 10.52 kg, reflecting a sustained upward trend [1]. The growing demand for chicken has accelerated the development of large-scale and intensive broiler farming. Under such conditions, increasing stocking density has raised both management difficulty and the risk of disease transmission, making the timely identification and removal of dead chickens essential for maintaining farm biosecurity and economic efficiency. For example, an experimental farm operated by Shuyang Zhongke Poultry Breeding Co., Ltd. in Suqian, Jiangsu Province, houses 28,000 white broilers and records 10–20 deaths per day, highlighting the practical need for accurate dead chicken detection in large-scale farming systems.

Conventional manual inspection is increasingly unable to meet the demands of modern poultry farming. In poultry houses containing 30,000–50,000 broilers, a complete inspection typically requires 1–2 workers and approximately 4 h. This task becomes even more difficult under low-light conditions, where it is more challenging and time-consuming to identify dead chickens. Therefore, replacing manual inspection with automated monitoring has become an urgent need in the poultry industry. This aligns with broader trends in intelligent systems for agriculture and beyond, where automated, data-driven solutions are increasingly critical. For instance, the development of robust and privacy-preserving intelligent systems, such as those based on adaptive federated learning [2], and the application of augmented intelligence for critical infrastructure like emergency vehicle trajectory prediction [3] demonstrate the power of integrating advanced computational models to solve real-world challenges. Our work applies a similar philosophy to poultry health management. Such a strategy can improve detection efficiency and accuracy while also helping to interrupt disease transmission, reduce farming risks, and enhance food safety [4,5]. In this context, deep-learning-based object detection provides an important technical route for intelligent dead chicken detection.

### 1.2. Deep Learning Approaches for Dead Chicken Detection

Compared with traditional image recognition methods, deep learning has shown strong generalization ability in image analysis because of its powerful feature extraction capability. With its rapid development, deep learning has been increasingly applied in agricultural production, especially in visual monitoring and target detection tasks. Existing dead chicken detection methods can generally be divided into two categories: two-stage methods and one-stage methods. Two-stage methods, represented by Mask R-CNN [6] and Faster R-CNN [7], usually achieve high detection accuracy by first generating candidate regions and then performing classification and localization. However, their inference speed is often insufficient for real-time applications. By contrast, one-stage methods, such as the YOLO series [8,9] and SSD [10], integrate classification and localization into a single stage, offering higher detection speed and better suitability for real-time deployment. In addition, their relatively lightweight architectures make them more suitable for mobile and embedded platforms. With continuous algorithmic advances, one-stage methods have also achieved substantial improvements in accuracy and therefore show broad potential for poultry farm monitoring and disease prevention.

Recent studies have explored dead chicken detection from different perspectives. Pei et al. improved YOLOv8n by incorporating CSPHet, SEAM, and DySample, thereby enhancing detection accuracy and real-time performance in complex environments. Yang et al. [11] optimized YOLOv7 and demonstrated its ability to identify dead chickens rapidly and accurately in caged environments. Ma et al. [12] developed an autonomous inspection robot for caged layer houses to enable automatic dead chicken detection and alarm generation. Zheng et al. [13] proposed a machine-vision-based monitoring system for vertically caged laying hens, which could identify dead chickens in real time. Jiang et al. [14] combined robotics, infrared thermal imaging, and image processing to design a temperature-based dead chicken recognition algorithm. Although these studies differ in research object and application scenario, they provide valuable references for poultry visual monitoring and automated dead chicken inspection.

Nevertheless, existing methods still have clear limitations when applied to practical large-scale caged broiler houses. Most studies have been conducted under relatively ideal conditions with sufficient illumination and low flock density, making them difficult to generalize to real farming environments. RGB-based methods often show unstable performance under severe occlusion and nighttime low-light conditions. Although infrared thermal imaging can capture temperature-related features, methods relying solely on infrared information are easily affected by ambient temperature fluctuations and background thermal noise, which may lead to false detections. In addition, such methods usually require relatively expensive hardware and often lack effective fusion with visible-light information. Therefore, how to effectively exploit the complementary advantages of RGB and infrared data while maintaining detection accuracy, real-time performance, and deployability remains a key issue in dead chicken detection research.

In practical farming scenarios, dead chicken detection faces three major challenges. First, high-density flocking leads to severe mutual occlusion, which greatly increases the difficulty of target recognition. Second, low-light conditions are common at night, making stable detection difficult when relying only on RGB images. Third, variations in growth stage, posture, and imaging distance result in significant scale differences among targets, making multi-scale recognition difficult for models with fixed convolution kernels. At the same time, real applications impose strict requirements on both inference speed and computational efficiency, since inspection robots must process images in real time, whereas edge devices such as Jetson Nano have limited computing resources. These constraints require dead chicken detection models to achieve high accuracy, high real-time performance, and lightweight deployment simultaneously. The details are shown in Table 1.

While existing studies have explored RGB-IR fusion for generic object detection or used single-modality vision for poultry monitoring, they often fall short in the specific context of caged broiler farms. Most RGB-IR fusion methods are designed for autonomous driving or surveillance and do not account for the unique challenges of severe occlusion from cage bars and high flocking density. Conversely, existing poultry monitoring studies primarily rely on a single modality (RGB or IR), making them susceptible to either poor lighting or thermal noise. Therefore, how to effectively exploit the complementary advantages of RGB and infrared data while maintaining detection accuracy, real-time performance, and deployability remains a key issue in dead chicken detection research. Our proposed method differs by introducing a dynamic hybrid convolution feature fusion module (DynMixC3k2Conv) and an occlusion-aware detection head (MultiSEAMHead) specifically designed to tackle the intertwined issues of multi-scale feature variation and occlusion within a dual-modal fusion framework.

To address these challenges, this study proposes a dual-modal dynamic hybrid convolution feature fusion detection method based on an improved YOLO11 framework, termed YOLO11-DualDynConv-FF. The proposed method introduces a dual-modal fusion architecture to simultaneously process RGB and infrared images, thereby combining visible-light shape and contour information with infrared temperature distribution information to improve detection robustness under low-light conditions. In addition, a dynamic hybrid convolution feature fusion module (DynMixC3k2Conv) is designed to adaptively adjust convolution kernel weights, enhancing multi-scale feature extraction and cross-layer feature fusion. In this way, the proposed method improves the recognition of dead chickens with different sizes and postures while reducing computational redundancy, providing an effective technical solution for intelligent dead chicken detection in complex farming environments.

Additionally, we design an occlusion-aware detection head, termed MultiSEAMHead, which incorporates an attention mechanism to enhance local discriminative features and suppress irrelevant background interference. By strengthening the representation of partially visible targets, this module effectively alleviates false positives and missed detections caused by severe occlusion and complex backgrounds. Experimental results show that the proposed model outperforms the original YOLO11 and several mainstream detection models in terms of precision, recall, F1-score, and mAP@0.5. At the same time, the proposed method maintains relatively low parameter count and computational cost, achieving accurate and robust dead chicken detection in complex farming environments. These characteristics make it a practical technical solution for automated inspection and intelligent farm management. The main contributions of this study are summarized as follows:

- We propose a dual-modal fusion architecture that improves adaptability under low-light conditions by jointly using RGB and IR images as inputs. This design combines the shape and contour information from visible-light images with the thermal distribution information from infrared images, thereby alleviating the performance degradation of single-modal RGB detection in nighttime or poorly illuminated environments.
- We develop a dynamic hybrid convolution feature fusion module to enhance multi-scale feature extraction and cross-layer feature interaction. By adaptively adjusting convolution kernel weights and integrating multi-scale convolutional features, the proposed module enables the model to better capture dead chicken targets with different sizes and postures, thus overcoming the limited scale adaptability of conventional fixed-kernel convolutions in complex scenes.
- We introduce an occlusion-aware detection head (MultiSEAMHead) to improve the recognition of partially occluded targets. By enhancing local feature representation and suppressing background interference, this module strengthens the detection of dead chickens under severe occlusion and complex background conditions, thereby reducing both missed detections and false detections.

## 2. Materials and Methods

### 2.1. Experimental Environment

**Figure 1:**
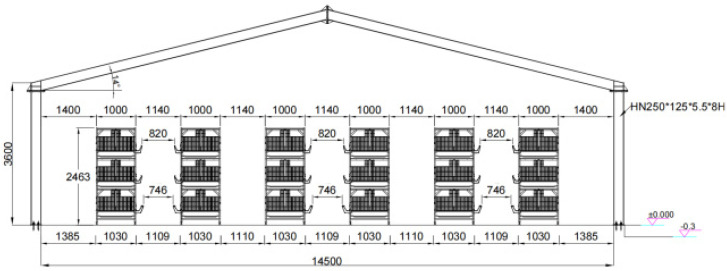
Frame diagram of henhouse.

This study was conducted at Yike Farm in Shiling Town, Suqian City, Jiangsu Province, to investigate the growth performance of white broilers under specific rearing conditions. White broilers are widely used in modern poultry production because of their rapid growth and high feed efficiency. Figure 1 shows the detailed structure of the experimental poultry house, which measured 90 m in length, 14.5 m in width, and 3.5 m in height. The internal layout featured a 6-row, 3-tier H-shaped stacked cage system. The interior design prioritized efficient use of space and operational convenience, with aisles between cage rows set at 90 cm to facilitate husbandry management and the operation of automated equipment. Each cage measured 1350 mm in length, 1000 mm in width, and 760 mm in height, accommodating 25 to 27 white-feathered broilers. Each tier housed 60 cages, and the entire facility accommodated approximately 28,000 white broilers. The H-shaped stacked cage system not only maximized space utilization but also facilitated automated management, significantly improving operational efficiency while reducing labor costs. In addition, this configuration may help improve flock welfare, reduce the risk of disease transmission, and provide a healthier and more suitable growth environment for broilers.

### 2.2. Dataset Collection and Construction

This study employed a FLIR T560 handheld infrared thermal imager for multimodal data acquisition. As an integrated device, its visible-light and infrared sensors were factory-calibrated and aligned at the hardware level, providing good initial spatial correspondence between the two modalities. The camera was configured with an imaging resolution of 640 × 480 pixels, a sampling frequency of 30 Hz, and a temperature measurement range of −20 °C to 120 °C. During the experiments, the device was mounted on a fixed bracket at a distance of 400–500 mm from the chicken cages, with the lens positioned at an angle of 20–25° relative to the horizontal plane. This setup ensured effective coverage of the target area while reducing optical distortion. The system synchronously captured infrared thermal information and visible-light images, and the corresponding RGB and infrared videos were stored in MP4 format, thereby ensuring the accuracy and reliability of the collected data.

**Figure 2:**
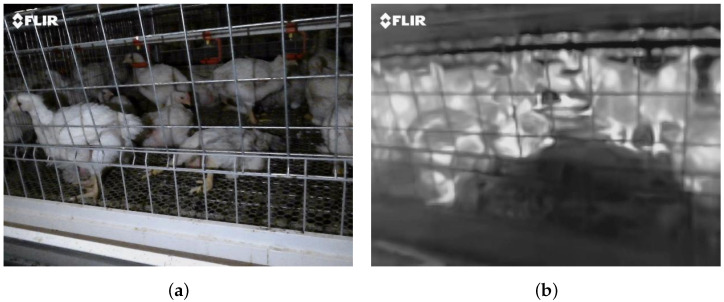
Sample dataset: (**a**) original RGB image; (**b**) corresponding infrared image.

Data collection was carried out from October 2024 to January 2025, yielding 50 valid paired video samples. Each sample consisted of one RGB video and one infrared video. For subsequent processing, a dedicated Python script was developed to extract key frames from the videos. Considering target motion characteristics and computational resource constraints, a down-sampling strategy with a 15-frame interval was adopted; that is, one frame was extracted every 15 frames (Figure 2). This strategy reduced the data volume while preserving effective motion information, thereby improving processing efficiency. To account for possible temporal misalignment of up to ±3 frames between the two modalities, manual verification was performed to correct cross-modal frame pairing and ensure accurate image correspondence. Ultimately, 2299 valid RGB-infrared image pairs were obtained, with each pair consisting of one RGB image and one infrared image. During the dataset construction, dead chickens in all image pairs were manually annotated using bounding boxes by PhD students specializing in poultry science, resulting in approximately 8000 annotated dead chicken instances. To ensure annotation accuracy, the annotated dataset was subsequently reviewed and checked by Master’s students in the field of poultry science. The inspection results showed a high degree of Intersection over Union (IoU) consistency between the two annotations, indicating strong reproducibility and accuracy of the labeling results. The distribution of dead chicken instances in the dataset is relatively balanced, meeting the basic requirements for training samples in deep learning models. Finally, the dataset was divided into training, validation, and test sets in a ratio of 7:2:1 to support subsequent deep learning model training and performance evaluation.

The research data were collected from a single farm and a single breed, which presents limitations in assessing the generalizability of the model. To evaluate the model’s performance under these conditions, two strategies were implemented. First, the dataset was divided into training, validation, and test sets based on date and cage row, ensuring that the test data, although from the same farm, consisted entirely of unseen images, thereby simulating a within-farm deployment scenario. Second, the test data were further categorized into three challenging scenarios, namely, low illumination, severe occlusion, and complex background, to evaluate model performance under specific conditions. Through this image attribute-based manual annotation and qualitative comparative analysis, the model’s performance variations under specific conditions could be examined, and its potential weaknesses in new environments could be identified.

To enhance the transparency of the dataset composition, Table 2 quantifies the distribution of image pairs across each major challenging scenario.

### 2.3. YOLO11 Network Model

YOLO11n is a lightweight object detection model in the YOLO11 series. Compared with larger variants such as YOLO11s and YOLO11x, YOLO11n achieves faster inference speed with fewer parameters and lower computational cost, making it more suitable for real-time applications under limited hardware resources.

**Figure 3:**
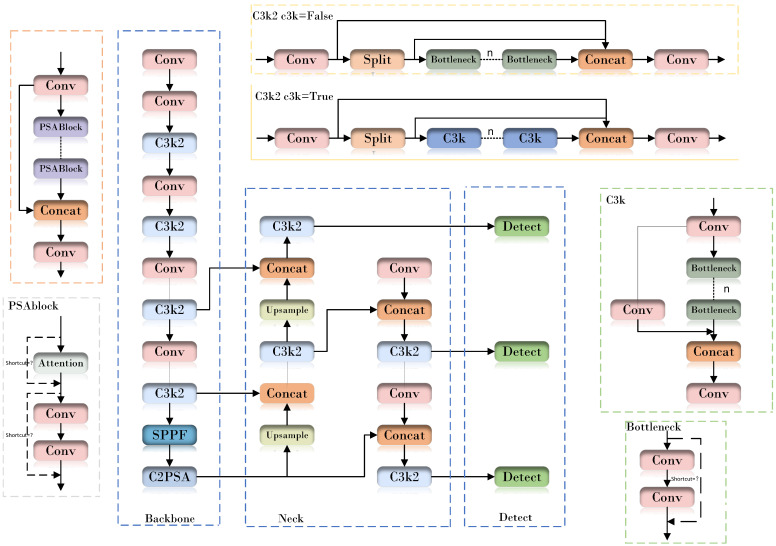
YOLO11n network structure diagram.

As shown in Figure 3, the YOLO11n architecture consists of four parts: the input layer, backbone, neck, and detection head. The input stage performs image preprocessing and adaptive resizing, while data augmentation strategies such as mosaic augmentation and cropping are used to improve data diversity and model robustness.The backbone network includes Conv, C3k2, SPPF, and C2PSA [15] modules for hierarchical feature extraction. Among them, C3k2 is developed based on C2f and integrates C3k and Bottleneck configurations, thereby balancing computational efficiency and feature extraction capability. When the C3k configuration is enabled, additional convolution operations are introduced to strengthen local feature extraction in complex scenes. The SPPF module enlarges the receptive field and enhances multi-scale contextual fusion, whereas C2PSA introduces position-sensitive attention to further improve feature representation. The neck network combines PAN and FPN [16] to fuse feature maps at different levels and scales. The detection head adopts a decoupled and anchor-free design, enabling efficient classification and localization across multiple scales.

### 2.4. Dead Chicken Detection Model Design

#### 2.4.1. YOLO11-DualDynConv-FF

In real breeding environments, various interference factors, such as uneven illumination, dense chicken flocks, and complex backgrounds, pose great challenges to dead chicken detection. Traditional manual inspection methods usually suffer from high labor costs and low efficiency when dealing with such complex scenarios, making it difficult to satisfy the current demand for accurate and efficient dead chicken detection in modern poultry production.

To address these issues, this study proposes targeted improvements based on the YOLO11 model. The potential limitations of the original model in complex breeding scenarios were systematically analyzed, and corresponding improvement strategies were developed. In terms of feature extraction, the feature representation capability of the network was enhanced to better capture key characteristics of dead chickens in complex environments. Meanwhile, the detection head was optimized to improve the detection of dead chickens with different sizes and postures. In addition, new auxiliary modules were introduced to further strengthen the model’s adaptability and robustness under complex background conditions.

**Figure 4:**
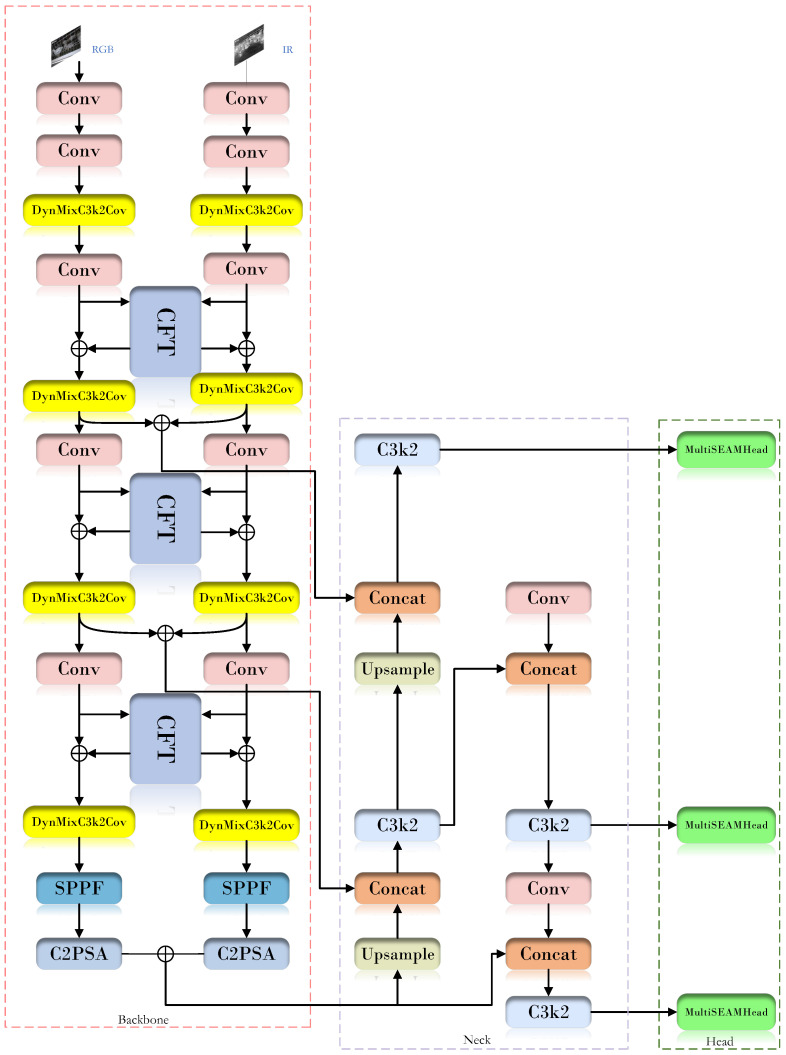
YOLO11-DualDynConv-FF structure.

After these improvements, the proposed model showed superior performance in handling complex situations in real chicken farms. Experimental results demonstrated that the improved model achieved significantly better detection performance in the dead chicken detection task, with a 12.1% improvement over the baseline model, while the false detection rate and missed detection rate were both reduced. The detailed network architecture is shown in Figure 4, which illustrates the connections among the modules and the key improvement points.

#### 2.4.2. Dual-Modal Fusion Network Framework

**Figure 5:**
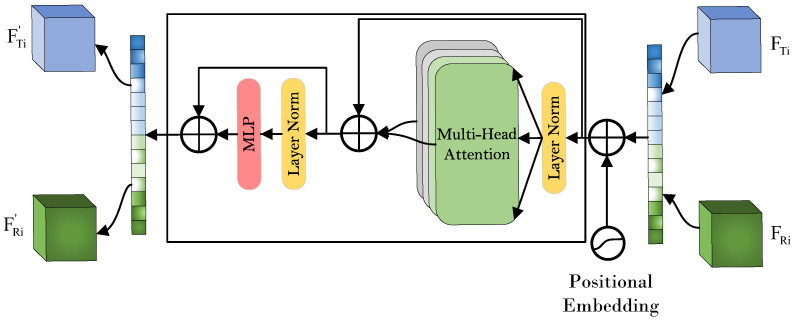
CFT module.

In the dead chicken detection task, IR and RGB images reflect different types of physical information about caged broilers. IR images provide temperature distribution information of chickens and cages, whereas RGB images present visual cues such as body shape and feather color [17]. If these two modalities are used independently, the model may learn only limited feature representations, thereby increasing the risk of misclassification and reducing detection performance. To address this issue, this study proposes a dual-modal fusion network framework. Specifically, the framework introduces a Cross-Modality Fusion Transformer (CFT) module [18], which enables the model to process information from both modalities simultaneously. By integrating complementary information from IR and RGB images, the proposed method can perform dead chicken detection more accurately and efficiently. The overall network architecture is shown in Figure 5.

The CFT module is built upon the Transformer architecture. Unlike traditional convolutional neural networks (CNNs), the Transformer is capable of capturing long-range dependencies and modeling global contextual information. In CNNs, convolution operations can be regarded as non-fully connected graphs with local receptive fields, whereas the self-attention mechanism in the CFT module can be viewed as a fully connected graph that facilitates global information learning. Leveraging this property, the CFT module performs hybrid fusion during the feature extraction stage, rather than relying on simple early input-level fusion or late decision-level fusion. This design enables dynamic integration of global contextual information from different modalities at multiple network levels.

Specifically, the core integration strategy of the CFT module is a dynamic interaction mechanism based on self-attention [19]. Through a carefully designed attention map, the module simultaneously performs intra-modal fusion and inter-modal fusion. Intra-modal fusion refers to the enhancement of internal features within the same modality, such as RGB or IR, thereby improving the representational capability of that modality. Inter-modal fusion refers to the complementary interaction between features from different modalities, which helps capture potential correlations between RGB and IR information. The dynamic nature of the module lies in the fact that the fusion weights are not fixed; instead, they are generated adaptively in real time according to the input content through attention calculations. In this way, the network can automatically determine where and to what extent information from different modalities should be fused. Therefore, the CFT module can effectively handle the complex interactions between RGB and IR modalities within a unified and adaptive framework, thereby significantly improving the performance of multispectral target detection.

#### 2.4.3. Dynamic Hybrid Convolution Feature Fusion Module

In dead chicken detection tasks based on IR and RGB fusion, the limitations of traditional CNNs are particularly evident. Due to significant variations in the morphology, size, and location of dead chickens in chicken coop environments, a single fixed-size convolution kernel cannot effectively capture the key characteristics of dead chickens at different scales, which leads to the following problems:

- For small dead chicken targets, large convolution kernels may fail to accurately capture fine-grained details; for large targets, small convolution kernels may be insufficient to extract global contextual information, thereby affecting detection accuracy.
- The fused feature maps of IR and RGB images contain rich semantic information at multiple levels. However, traditional CNNs often cannot efficiently integrate such information, resulting in insufficient utilization of the relationships between shallow and deep features, which in turn affects target detection performance in complex chicken coop environments.
- Due to the complexity and variability of chicken house environments [20], fixed convolution operations lack flexibility and cannot adaptively adjust the convolution strategy according to the characteristics of the input image. As a result, they struggle to meet the detection requirements of dead chickens under varying lighting and occlusion conditions, thereby reducing the robustness and adaptability of the model.
- The use of fixed convolution kernels may introduce unnecessary computational redundancy [21], leading to inefficient training and real-time inference when processing large-scale chicken coop image datasets. This increases memory consumption and computational latency, which is unfavorable for rapid detection and real-time feedback in practical applications.

**Figure 6:**
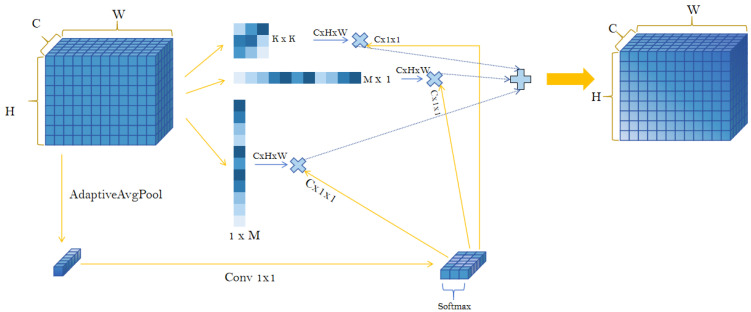
DynamicInceptionDWConv2d module structure diagram.

To address the above issues, we introduced a dynamic hybrid convolution feature fusion module, namely DynamicInceptionDWConv2d. Based on the C3k2 structure, we further designed the DynMixC3k2Conv module to provide a flexible and efficient convolution architecture for dead chicken detection in complex breeding scenarios. As shown in Figure 6, the proposed module achieves this goal through the following designs. First, a dynamic convolution kernel weighting mechanism [22] is introduced to assign adaptive weights to different convolution kernels, allowing the network to dynamically adjust kernel usage according to the requirements of the input feature maps. This mechanism overcomes the limitations of traditional fixed convolution kernels and enables the convolution operation to adapt to input features in each forward pass, thereby effectively capturing multi-scale and diverse feature information. Second, by combining multi-scale convolution kernels, information can be extracted and flexibly fused at different scales, enabling the network to capture fine-grained multi-scale features rather than relying on a single fixed-size convolution kernel. This significantly improves the adaptability of the model to complex scenes. In addition, by multiplying the outputs of different convolutional layers with the corresponding dynamic weights, effective cross-layer information fusion is achieved. Within each module, the convolutional feature maps are added to the residual of the input feature map, which preserves the effective transmission of both low-level and high-level features throughout the network. This not only enables the model to make full use of multi-level information but also mitigates the vanishing gradient problem and feature loss caused by excessive network depth.

**Figure 7:**
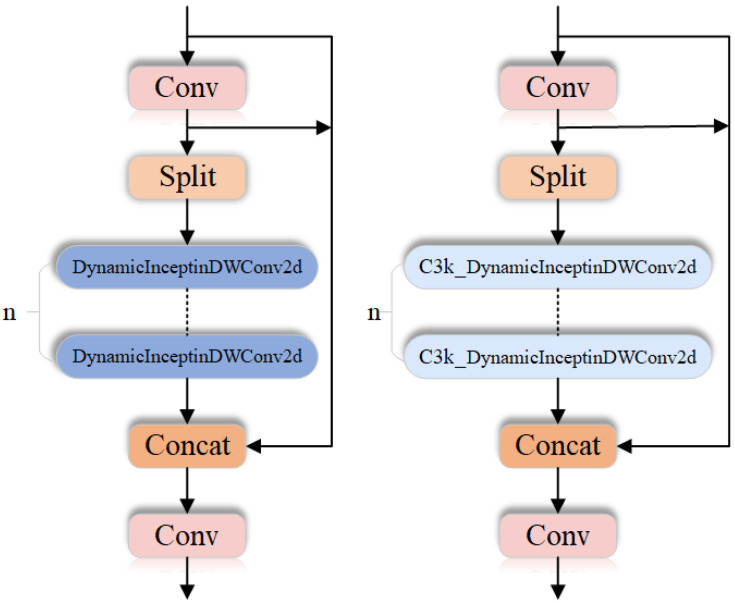
Structure diagram of DynMixC3k2Conv.

In terms of computational efficiency, the DynMixC3k2Conv module (shown in Figure 7) adopts depthwise convolution, which significantly reduces the number of parameters and computational cost while still allowing detailed processing of the input feature maps. Through dynamic adjustment of convolution kernels, the model improves its performance on diverse inputs while reducing unnecessary computational redundancy.

#### 2.4.4. Occlusion Perception Module

**Figure 8:**
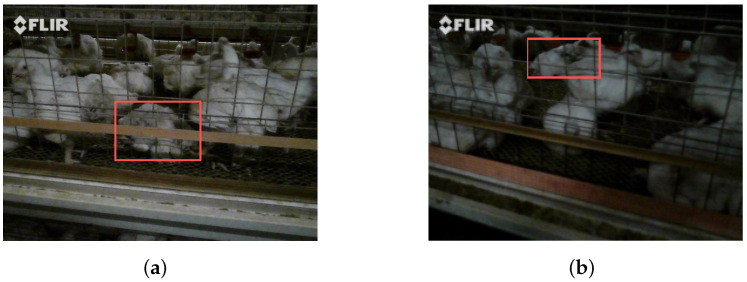
Cage occlusion: (**a**) the cage occlusion of the target area, (**b**) the overlap of dead chickens and normal chickens.

**Figure 9:**
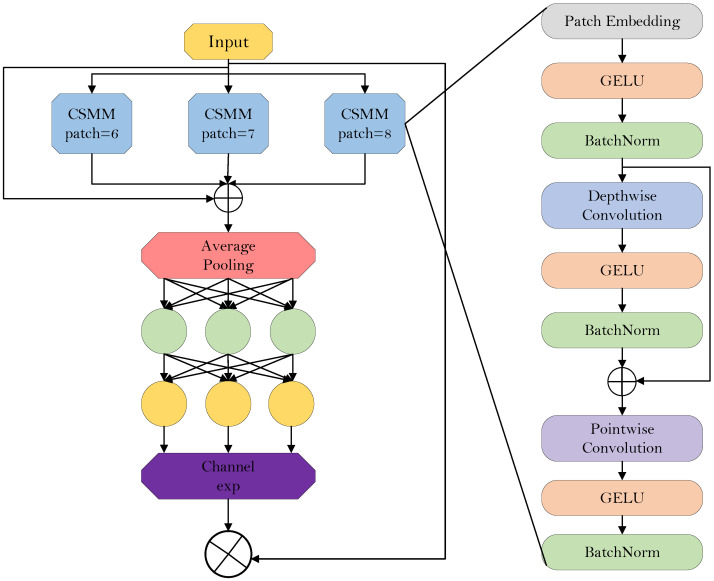
SEAM module structure diagram.

In breeding cages, chickens may be densely crowded and overlapped, causing some individuals to be occluded by others and making them difficult to identify accurately [23,24]. In addition, the breeding environment is complex and variable, containing various background elements such as the ground, fences, and troughs, which may share visual similarities with chickens in terms of color, texture, and other characteristics [25]. As shown in Figure 8, these factors interfere with the accurate detection of dead chickens. To address these challenges, this study draws inspiration from the design of the SEAM module (shown in Figure 9), further redesigns the detection head, and proposes the MultiSEAMHead module.

In dead chicken detection tasks, the SEAM module demonstrates significant advantages through multi-scale feature enhancement and background interference suppression. Its core design is inspired by the idea of patch embedding and combines dynamic fusion of features at different scales to accurately enhance local details of chicken targets, such as feather texture and posture anomalies, in complex breeding scenarios. Meanwhile, through the lightweight combination of depthwise separable convolution [26] and pointwise convolution, spatial features are processed hierarchically and channel information is optimized, enabling efficient capture of key discriminative features of dead chickens at multiple scales. The GELU activation function and batch normalization (BatchNorm) embedded in the module further stabilize the training process and enhance the robustness of the model against interference factors such as illumination variations and occlusion. In addition, the SEAM module significantly suppresses the influence of background regions, such as feed, cages, and dynamic lighting, through an attention-like screening mechanism, thereby reducing the risk of false detections caused by similar backgrounds and allowing the detector to focus more accurately on target regions under limited computational resources. This capability enables the module to resist background noise in complex and variable breeding environments and rapidly localize weakly salient targets, such as fallen dead chickens, ultimately improving detection accuracy and real-time performance and providing a highly adaptive solution for dead chicken detection.

**Figure 10:**
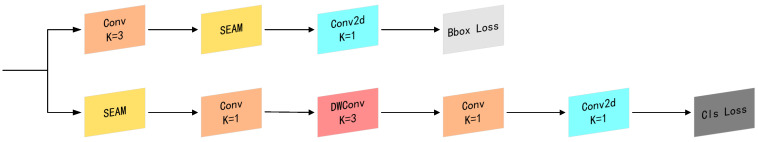
MultiSEAMHead Module.

The MultiSEAMHead module proposed in this study further extends and optimizes these advantages. It can more effectively address the occlusion problem caused by densely crowded and overlapped chickens. Through more refined feature enhancement and fusion strategies, the module can extract latent feature information from occluded chickens and improve recognition performance for occluded dead chickens. At the same time, in terms of multi-scale feature extraction, the MultiSEAMHead module incorporates richer multi-scale feature fusion strategies to ensure accurate detection of dead chickens with different sizes and postures. Whether subtle local characteristics of dead chickens or overall body shape over a larger spatial range, these features can be effectively captured and identified. As a result, the proposed module enables more efficient and accurate dead chicken detection in complex breeding environments, thereby improving the practicality and reliability of the detection model. Figure 10 shows the structural diagram of the MultiSEAMHead module.

### 2.5. Training Environment and Evaluation Indicators

#### 2.5.1. Experimental Environment and Parameter Settings

The experimental platform consisted of a Windows operating system, an NVIDIA RTX 5070 Ti GPU with 16 GB of memory, and an Intel Core i7-13700K CPU. The deep learning software environment included PyTorch 2.2.0, CUDA 11.8, and Python 3.10. After multiple rounds of experiments and hyperparameter optimization, the training settings used in this study were determined, and the detailed parameter configurations are listed in Table 3.

#### 2.5.2. Evaluation Indicators

In this study, to comprehensively evaluate the performance of the model, we adopted several key metrics, including Precision, Recall, F1-score, and mean Average Precision (mAP). Among them, mAP is a core evaluation metric [27,28], which is widely used to quantify the detection performance of a model in multi-class object detection tasks. It is obtained by first plotting the precision–recall (P–R) curve; then calculating the area under the curve for each category, namely the Average Precision (AP); and finally averaging the AP values over all categories to obtain the mAP. The calculation of Precision, Recall, and AP is based on the statistical analysis of True Positives (TPs), False Positives (FPs), and False Negatives (FNs). By jointly considering these metrics, the performance of the model can be comprehensively assessed while also providing deeper insight into its behavior in different application scenarios. The formulas for Precision (*P*), Recall (*R*), mAP, and F1-score are given in Equations (1)–(4).

(1) $$P=\frac{\mathrm{TP}}{\mathrm{TP}+\mathrm{FP}}$$

(2) $$R=\frac{\mathrm{TP}}{\mathrm{TP}+\mathrm{FN}}$$

(3) $$\mathrm{mAP}=\frac{1}{N}\sum_{i=1}^{N}\mathrm{AP}\left(i\right)$$

(4) $$F1\text{-}\mathrm{score}=\frac{2\mathrm{TP}}{2\mathrm{TP}+\mathrm{FN}+\mathrm{FP}}$$

## 3. Results

In this section, we will focus on the performance results of the proposed model.

### 3.1. Ablation Experiment

As shown in Table 4, to verify the advantages of the improved method for dead chicken detection in cage-raising systems, this study systematically designed ablation experiments and evaluated eight different configurations under a unified hardware and parameter setting. The individual effects of the CFT, DynMixC3k2Conv, and MultiSEAMHead modules were first assessed, resulting in significant increases in mAP@0.5 of 9.3%, 5.6%, and 7.0%, respectively. However, the integration of these modules revealed a more complex interaction. Some dual-module combinations (e.g., CFT + MultiSEAMHead) performed worse than certain single-module configurations, which may suggest potential functional overlap or optimization interference. A deeper analysis of the CFT + MultiSEAMHead combination reveals a possible reason. The CFT module excels at global, cross-modal feature fusion via a Transformer architecture, while the MultiSEAMHead, with its attention-based mechanism, also focuses on enhancing local discriminative features to handle occlusion. The degradation in performance (mAP@0.5 dropping from 77.6% for CFT alone to 74.7% for the combination) suggests that these two attention-heavy modules may create redundant feature representations, leading to overfitting on certain patterns and interfering with gradient flow during optimization. This indicates that while each module is effective independently, their direct combination requires careful balancing to avoid competitive rather than complementary learning. In contrast, the full integration of all three modules exhibited a synergistic effect. In the final unified architecture, namely YOLO11-DualDynConv-FF, the three modules complemented each other, where CFT improved cross-modal feature fusion, DynMixC3k2Conv enhanced local feature modeling, and MultiSEAMHead strengthened multi-scale perception. As a result, the proposed model achieved a peak mAP of 80.1%, representing an 11.8% improvement over the baseline and outperforming all single-module and dual-module configurations. These results indicate that the proposed method identifies an effective complementary module combination and achieves substantial performance gains while maintaining manageable model complexity.

**Figure 11:**
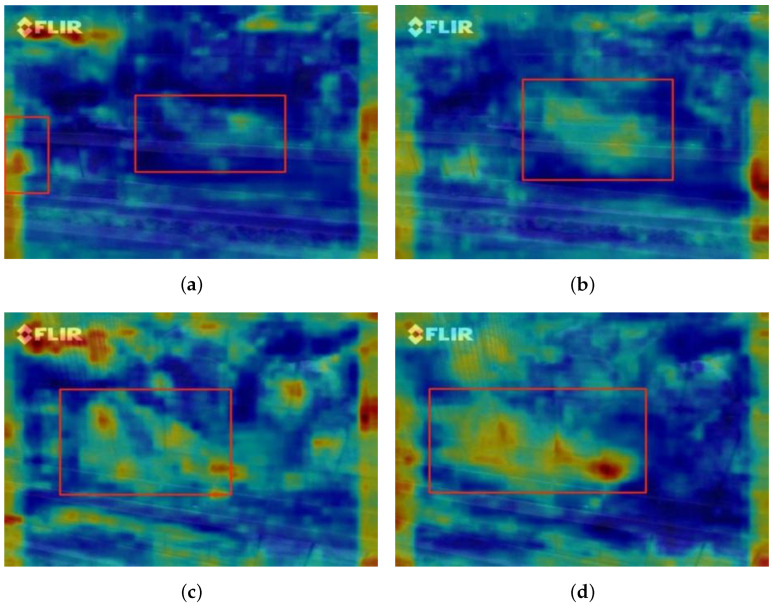
Comparison of feature extraction results before and after introducing the module. Panels (**a**,**b**) form one group, where panel (**a**) shows the result before adding the module and panel (**b**) shows the result after adding the module. Panels (**c**,**d**) form another group, where panel (**c**) shows the result before adding the module and panel (**d**) shows the result after adding the module.

To further verify the effectiveness of the dynamic hybrid convolution feature fusion module, a feature heatmap visualization experiment was conducted [29,30]. By comparing the feature heatmaps of the network before and after introducing the module, the differences in feature extraction behavior on the input images can be visually analyzed. This qualitative analysis provides intuitive evidence of the enhancement in feature extraction capability brought by the proposed module. Figure 11 shows the comparison of feature extraction results before and after the module is introduced.

As shown in the heatmaps, after the dynamic hybrid convolution feature fusion module is introduced, the network exhibits stronger and more concentrated responses in the target regions. In the visualization, warmer colors indicate stronger feature responses. Compared to the baseline network, the model equipped with the proposed module shows a more prominent activation in the relevant target areas, suggesting an improved representation of characteristics for the dead chicken regions. This comparative analysis further supports the feasibility and effectiveness of the proposed module, and the visual observations are consistent with the quantitative improvements observed in the ablation experiments.

### 3.2. Comparative Experiments Under Different Models

To ensure a fair comparison, dual-modal variants were implemented for multiple baseline models, including YOLOv5, YOLOv8, YOLOv12 [31], Hyper-YOLO [32], DETR [33], YOLO11, and the proposed YOLO11-DualDynConv-FF model (denoted as “Ours” in the table). Table 5 shows the detailed experimental results of each model in single-modal and dual-modal states, including parameter quantity (Param.), computational quantity (GFLOPs), average accuracy (mAP), accuracy (P), recall (R) and F1 score.

Experimental results show that the dual-modal strategy significantly improved detection performance across different models compared with their single-modal counterparts. For example, mAP@0.5 increased from 69.0% to 75.1% for YOLOv5, from 67.4% to 73.6% for YOLOv8, and from 66.6% to 77.8% for YOLOv12. Among all compared models, the proposed YOLO11-DualDynConv-FF achieved the best overall performance, with mAP@0.5 reaching 80.1%, mAP@0.5:0.95 reaching 65.1%, precision reaching 92.6%, and F1-score reaching 0.85.

This dual-modal strategy significantly improves the detection performance of all models compared to their single-modal counterparts. Among the models compared, YOLO11-DualDynConv-FF achieves the best overall performance, with an mAP@0.5 of 80.1%, a precision of 92.6%, and an F1-score of 0.85. Although DETR achieves a higher recall (93.0%), its parameter count (66.32 M) and computational cost (194 GFLOPs) are substantially higher than those of the proposed model (3.17 M parameters, 6.85 GFLOPs). Compared to the dual-modal YOLO11 baseline, the proposed model shows only a slight increase in parameters (from 2.89 M to 3.17 M) and computational cost (from 6.34 to 6.85 GFLOPs) while improving mAP@0.5 by 2.5%. This method achieves a favorable balance between detection performance and computational efficiency, making it suitable for practical deployment.

**Figure 12:**
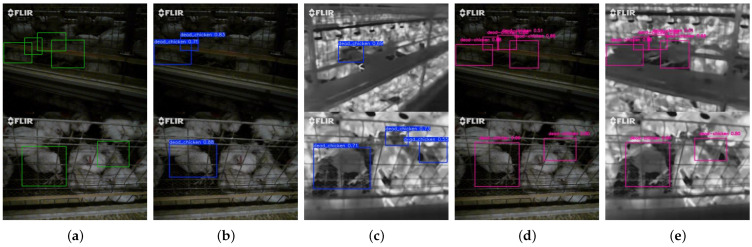
Comparison of detection results between YOLO11 and YOLO11-DualDynConv-FF. (**a**) Ground-truth annotations in the original images. (**b**) Detection results of YOLO11 on RGB images. (**c**) Detection results of YOLO11 on IR images. (**d**) Detection results of YOLO11-DualDynConv-FF on RGB images. (**e**) Detection results of YOLO11-DualDynConv-FF on IR images.

To further compare YOLO11-DualDynConv-FF with YOLO11, qualitative analysis was conducted from the perspectives of detection effect, detection accuracy, and dual-modal detection capability. Figure 12 presents a detailed comparison of the two models. The results show that YOLO11-DualDynConv-FF maintains more stable detection performance under both good and dim lighting conditions, with fewer false detections and missed detections. In addition, its ability to jointly utilize RGB and IR information further enhances detection efficiency and performance for dead chicken targets, especially in low-light environments.

**Figure 13:**
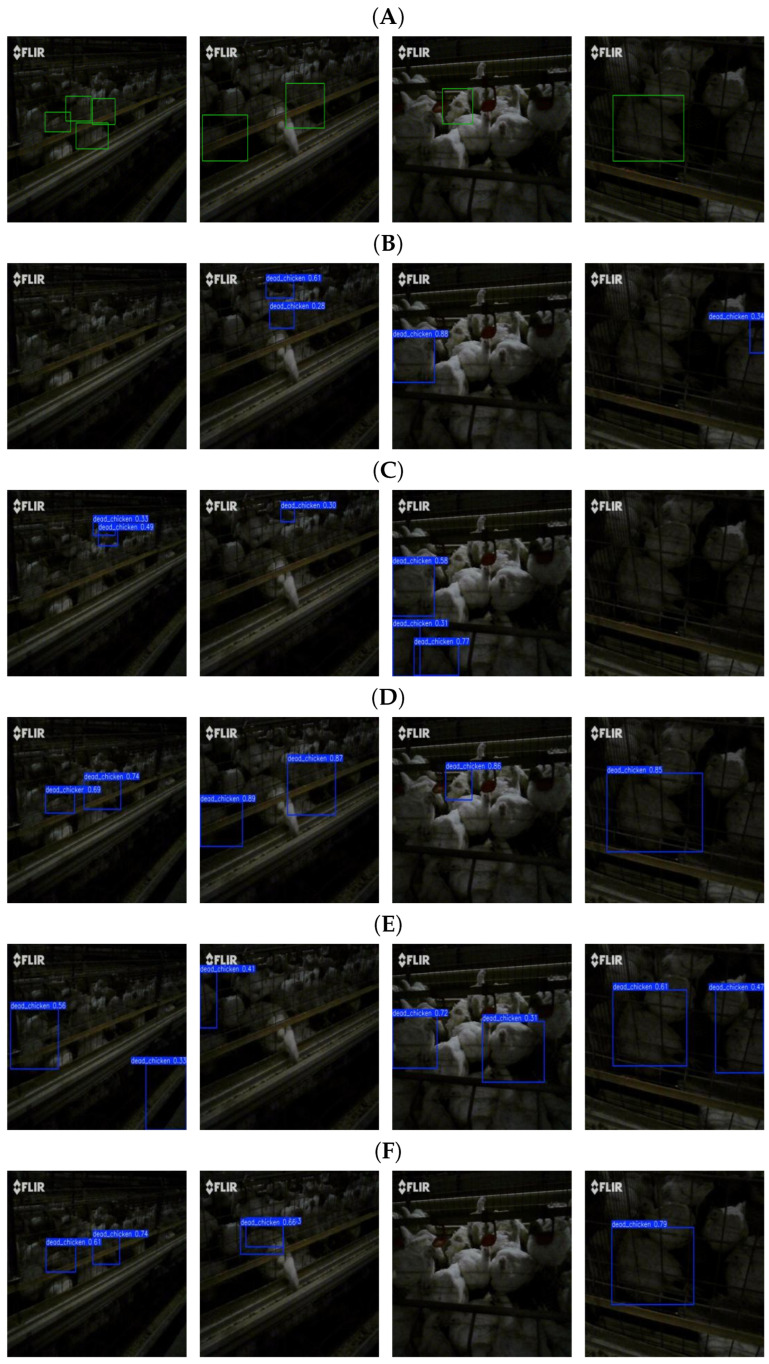

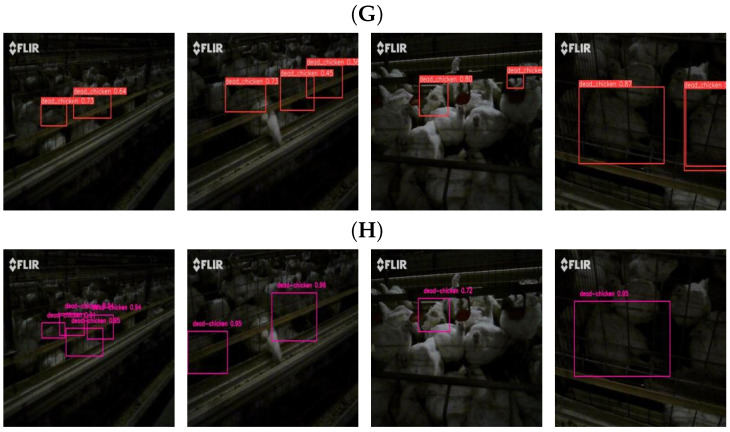
Qualitative comparison of experimental results under different challenging scenarios. Each column represents a different challenge: (Column 1) Complex background, (Columns 2 and 4) Occlusion, (Column 3) Low-light conditions. (**A**) Ground-truth annotations, (**B**) YOLOv5, (**C**) YOLOv8, (**D**) Hyper-YOLO, (**E**) YOLOv12, (**F**) YOLO11, (**G**) RT-DETR, (**H**) Proposed method.

Beyond the comparison with the baseline YOLO11 model, the proposed method was also qualitatively compared with YOLOv5, YOLOv8, YOLOv12, Hyper-YOLO, and RT-DETR. Figure 13 shows the detailed detection results. The results indicate that the proposed algorithm demonstrates strong detection performance not only against YOLO-based methods of the same level but also when compared with the strong-performing DETR model. In particular, under dim lighting conditions, the advantages of the proposed dual-modal detection algorithm become especially prominent. These experimental results further demonstrate the effectiveness and practical feasibility of the proposed method, providing useful methodological support for dual-modal detection in dead chicken monitoring tasks.

### 3.3. Model Deployment Comparison Experiment

**Figure 14:**
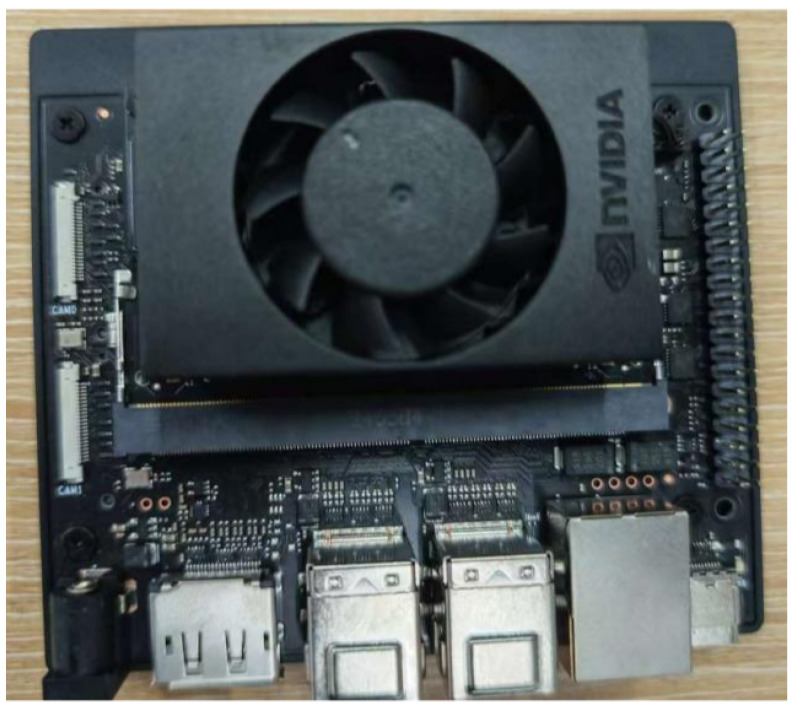
Jetson Orin Nano Photographs.

To verify the applicability of the proposed method in real-world scenarios, this study established a deployment pipeline for edge devices. The pipeline consists of three steps: converting the trained PyTorch model to ONNX format; loading the converted model onto the NVIDIA Jetson Orin Nano developer board; and performing inference using a Python script optimized for the Jetson architecture to complete detection on test images and record key performance metrics. The experiment aims to evaluate the deployment performance of different models on edge devices, providing a basis for subsequent integration into robotic platforms. All models involved in the comparison were trained and evaluated under the dual-modal framework. The edge deployment platform utilized the Jetson Orin Nano developer board, as shown in Figure 14, and TensorRT acceleration was not enabled during the experiments. Detailed experimental results are summarized in Table 6.

Experimental results showed that the proposed model performed well overall in the edge deployment environment, achieving an inference speed of 24 FPS, a preprocessing latency of 186 ms, and an end-to-end latency of 228 ms. Its inference speed was significantly higher than that of YOLOv5 (15 FPS), YOLOv8 (12 FPS), and DETR (9 FPS), while its preprocessing latency was also much lower than that of DETR (954 ms) and Hyper-YOLO (273 ms), indicating its potential for real-time inference on resource-constrained devices. Compared with YOLO11 (27 FPS) and YOLO12 (26 FPS, 164 ms preprocessing time), the proposed model still showed slight disadvantages in inference speed and preprocessing latency, and its end-to-end latency was also higher than that of YOLO11 (211 ms) and YOLO12 (202 ms), suggesting that further optimization in lightweight design and inference pipeline is needed.

Future work will focus on edge deployment. We plan to further reduce the computational overhead through model pruning and knowledge distillation, and to perform kernel-level adaptation and compilation optimization for common edge hardware platforms, thereby facilitating the efficient deployment of vision models in real-world scenarios.

## 4. Discussion

The ablation study results show that each module makes a unique contribution to detection performance. The CFT module leverages the Transformer architecture for dynamic cross-modal fusion, effectively integrating RGB and infrared information, achieving the largest individual mAP improvement of 9.3%. The DynMixC3k2Conv module employs dynamic convolution to adaptively capture multi-scale features, increasing mAP by 5.6%. The MultiSEAMHead module enhances occlusion handling through an attention mechanism, boosting mAP by 7.0%. Certain two-module combinations, such as CFT with MultiSEAMHead, led to performance degradation due to functional overlap, whereas the full integration of all three modules produced a synergistic effect, ultimately surpassing all other configurations.

Compared with existing methods, the proposed YOLO11-DualDynConv-FF achieves a better balance between accuracy and efficiency. Although DETR achieves a higher recall of 93.0%, its parameter count reaches 66.32 M and its computational cost reaches 194 GFLOPs, an order of magnitude higher than our model, which has only 3.17 M parameters and 6.85 GFLOPs. YOLOv12 and Hyper-YOLO also perform well, but our model achieves a better mAP@0.5 result with comparable or lower resource consumption. Qualitative comparisons further confirm that under low-light and occlusion conditions, the combination of dual-modal design and occlusion-aware attention enables the model to maintain stable detection in scenarios where single-modal RGB models fail.

Edge deployment results on the Jetson Orin Nano platform show that the model achieves an inference speed of 24 FPS and an end-to-end latency of 228 ms, outperforming YOLOv5, YOLOv8, and DETR in speed while maintaining high detection accuracy. However, preprocessing and end-to-end latencies are still slightly higher than those of YOLO11, indicating room for further optimization. Notably, prior studies have demonstrated that YOLOv11n achieves the fastest inference speed among YOLO series models, with a reported speed of 2.8 ms per frame (approximately 357 FPS) on high-performance hardware [34], highlighting the trade-off between detection accuracy and inference efficiency in practical deployment scenarios.

This study also has several limitations. The dataset comes from a single farm and a single breed. The current dual-modal fusion method does not adaptively weight the two modalities according to scene conditions. Although real-time performance meets most application requirements, further lightweight optimization is still needed. Moreover, the limited diversity of public computer vision datasets in precision livestock farming, particularly the lack of annotated data collected from diverse environments and animal breeds, remains a fundamental bottleneck for technology advancement [35].

Future research can be pursued in several directions: Expand the dataset to include more diverse farms, breeds, and environmental challenges. Explore adaptive fusion mechanisms that dynamically adjust modality weights based on input quality. Apply model compression techniques such as pruning, quantization, and knowledge distillation to reduce latency. Integrate the detection model into an autonomous inspection robot system to enable continuous, unattended poultry house monitoring.

## 5. Conclusions

This study proposes a method for dead chicken detection, named YOLO11-DualDynConv-FF, which is based on an improved YOLO11 framework and integrates dual-modal dynamic hybrid convolutional features. The method addresses the challenges of low detection efficiency, high labor costs, and difficulties in detection under dim lighting and occlusion conditions in caged broiler farms. A dual-modal fusion network architecture is introduced to process visible and infrared images simultaneously, significantly improving detection performance under low-light conditions. The dynamic hybrid convolutional feature fusion module enhances feature extraction capability while effectively reducing redundant information. An occlusion-aware module is also incorporated to better address occlusion issues in chicken cages.

Experimental results show that the proposed model outperforms the original YOLO11 model across key metrics, achieving a precision of 92.6%, recall of 79.0%, F1-score of 0.85, and mAP@0.5 of 80.1%. With only 3.17M parameters and an inference speed of 24 FPS on the Jetson Orin Nano, the model maintains a lightweight architecture [36,37] and competitive inference speed while delivering the above performance, offering a practical solution for intelligent dead chicken detection in caged poultry systems.

Although the current results are promising, the dataset is derived from a single farm and a single breed, and specifically lacks sufficient representation of different cage colors, lighting layouts, or chicken breeds. This limitation may lead to performance degradation when the model is deployed in unseen farm environments. The current dual-modal fusion approach does not adaptively weight the two modalities according to scene conditions, and while real-time performance meets most application requirements, further lightweight optimization is still needed. Future work will focus on expanding the dataset to include more farms, breeds, and environmental conditions, further optimizing the model for edge deployment, and integrating the detection system into an autonomous inspection robot to achieve full automation in farm management.

## Figures and Tables

**Table 1:** Key challenges addressed in this study.

Type of Challenge	Description of Specific Issues	Technical Requirements for This Study
Occlusion	High-density layered breeding leads to severe mutual occlusion among chickens, resulting in limited visible target regions, difficulty in feature extraction, and an increased missed detection rate.	The model should possess strong anti-occlusion capability, enabling it to identify targets from local visible regions while suppressing background interference.
Lighting	Under nighttime or insufficient illumination conditions in the henhouse, the quality of RGB images decreases significantly, image details are lost, and the performance of single visible-light models deteriorates sharply.	It is necessary to introduce complementary modalities, such as infrared imaging, to provide information independent of visible light and to achieve effective multi-modal feature fusion.
Multi-scale target variation	The body sizes of dead chicks and adult chickens differ considerably, and obvious scale variations may also appear within the same scene. Models with fixed receptive fields find it difficult to accurately detect targets of different sizes simultaneously.	The model should have dynamic and adaptive multi-scale feature fusion capability to accurately capture targets of different sizes.

**Table 2:** Distribution of dataset samples across primary challenging scenarios.

Scenario Category	Description of Specific Conditions	Number of Image Pairs
Low-Light	Images captured during evening/night or in shadowed areas, with poor RGB visibility.	831
Severe Occlusion	Dead chicken target is significantly obstructed by cage bars or other live chickens.	946
Complex Background	Background includes objects with similar color/texture to chickens.	522
Total Unique Image Pairs		2299

**Table 3:** Deep learning hyperparameters.

Parameter	Value
imgsz	640
batch	32
lr0	0.01
epoch	300

**Table 4:** Data comparison of ablation experiments.

Treatment	mAP@0.5/%	mAP@0.5:0.95/%	P/%	R/%	F1
YOLO11	68.3	54.8	90.6	74.0	0.68
YOLO11 + CFT	77.6	60.4	92.1	78.9	0.81
YOLO11 + DynMixC3k2Conv	73.9	59.3	90.8	75.7	0.72
YOLO11 + MultiSEAMHead	75.3	61.5	91.8	77.3	0.77
YOLO11 + CFT + DynMixC3k2Conv	79.8	63.2	92.5	79.1	0.84
YOLO11 + CFT + MultiSEAMHead	74.7	60.8	91.2	76.2	0.75
YOLO11 + DynMixC3k2Conv + MultiSEAMHead	72.4	58.4	90.7	74.5	0.69
YOLO11-DualDynConv-FF	80.1	64.5	92.6	79.0	0.85

**Table 5:** Comparison of detection performance between single-modal and dual-modal methods.

Models	Method	Param.	GFLOPs	mAP@0.5/%	mAP@0.5:0.95/%	P/%	R/%	F1
YOLOv5	Single modality	2.18 M	5.80	69.0	50.5	79.5	61.7	0.63
	Two Stream	3.67 M	9.80	75.1	56.8	88.1	73.4	0.72
YOLOv8	Single modality	2.68 M	6.80	67.4	48.8	80.4	64.2	0.66
	Two Stream	4.36 M	11.34	73.6	52.9	88.7	73.3	0.74
YOLO12	Single modality	2.51 M	6.0	66.6	48.2	70.3	59.4	0.69
	Two Stream	2.55 M	6.35	77.8	57.9	91.9	83.0	0.79
Hyper-YOLO	Single modality	3.95 M	10.97	72.5	52.4	76.7	62.0	0.69
	Two Stream	3.94 M	10.78	78.5	57.1	91.9	83.0	0.80
DETR	Single modality	72.94 M	234.7	70.2	49.6	90.7	95.0	0.72
	Two Stream	66.32 M	194	77.2	53.1	96.4	93.0	0.79
YOLO11	Single modality	2.58 M	6.32	68.3	50.6	90.4	74.0	0.68
	Two Stream	2.89 M	6.34	77.6	57.4	92.1	78.9	0.80
	Ours	3.17 M	6.85	80.1	65.1	92.6	79.0	0.85

**Table 6:** Comparison of deployment speed of different models.

Models	FPS	Preprocess	End-to-End Latency
YOLOv5	15	216 ms	283 ms
YOLOv8	12	235 ms	318 ms
YOLO12	26	164 ms	202 ms
Hyper-YOLO	19	273 ms	326 ms
DETR	9	954 ms	1065 ms
YOLO11	27	174 ms	211 ms
Ours	24	186 ms	228 ms

## Data Availability

As this is a proprietary dataset belonging to our collaborators, we are unable to disclose it at present.
